# EPAS-1 Mediates SP-1-Dependent FBI-1 Expression and Regulates Tumor Cell Survival and Proliferation

**DOI:** 10.3390/ijms150915689

**Published:** 2014-09-04

**Authors:** Xiaogang Wang, Peng Cao, Zhiqing Li, Dongyang Wu, Xi Wang, Guobiao Liang

**Affiliations:** 1 Department of Neurosurgery, Institute of Neurology, General Hospital of Shenyang Military Area Command, Shenyang 110016, China; E-Mails: xiaogangwangzy@163.com (X.W.); atocaopeng@hotmail.com (P.C.); zhiqianglizql@hotmail.com (Z.L.); wudychina@126.com (D.W.); 2 Institute of Neuroscience, Fourth Military Medical University, Xi’an 710032, China; E-Mail: wangzh@fmmu.edu.cn

**Keywords:** factor binding IST-1, specificity protein-1, endothelial pas domain protein-1, transcription factor, tumorigenesis

## Abstract

Factor binding IST-1 (FBI-1) plays an important role in oncogenic transformation and tumorigenesis. As FBI-1 is over-expressed in multiple human cancers, the regulation of itself would provide new effective options for cancer intervention. In this work, we aimed to study the role that EPAS-1 plays in regulating FBI-1. We use the fact that specificity protein-1 (SP-1) is one of the crucial transcription factors of FBI-1, and that SP-1 can interact with the endothelial pas domain protein-1 (EPAS-1) for the induction of hypoxia related genes. The study showed that EPAS-1 plays an indispensible role in SP-1 transcription factor-mediated FBI-1 induction, and participated in tumor cell survival and proliferation. Thus, EPAS-1 could be a novel target for cancer therapeutics.

## 1. Introduction

The POK (poxvirus and zinc finger domain) protein family member factor binding IST-1 (FBI-1, also named Pokemon, LRF or OCZF) was firstly identified as a factor that can bind specifically to the IST (inducer of short transcripts) element in the HIV-1 promoter region [1]. Currently, FBI-1 is well known by not only the function in regulating HIV infection but also the role it plays in adipogenesis, cell differentiation and oncogenesis [2,3,4]. FBI-1 acts as a transcription factor that regulates the expression of proteins such as c-myc, COMP (human cartilage oligomeric matrix protein) and extracellular matrix collagen type I [3,5,6]. It also acts as a transcription repressor that inhibits the expression of ADP-Ribosylation Factor (ARF) tumor suppressor, by which FBI-1 enhances the degradation of p53 and thus potentiates oncogenic transformation [7]. Recent literature has reported that FBI-1 also enhances tumorigenesis via several mechanisms, such as interrupting androgen receptor (AR) signaling or up-regulating phosphatidylinositol 3-kinase (PI3K)/Akt pathway [8,9]. Over-expression of FBI-1 could be detected in multiple cancers (such as bladder, breast and colon cancer) while FBI-1 knockout leaded to suppression of oncogenic transformation and tumor cell senescence and apoptosis [10]. In summary, FBI-1 plays an important role in tumorigenesis; it can be an eligible pharmaceutical target for cancer treatment. A deeper understanding of how proto-oncogene is regulated will be helpful in future therapeutic antitumor effects.

EPAS-1 is also known as hypoxia inducible factor 2α (HIF2α) which, as a nuclear transcription factor, plays an important role in cellular oxygen tension reduction responding [11]. EPAS-1 forms a heterodimer with the aryl hydrocarbon receptor nuclear translocator (ARNT) when cells have been stimulated with anoxic condition. The activation of endothelial pas domain protein-1 (EPAS-1) promotes this transcription factor to bind to the hypoxia response element (HRE) of target genes’ promoters [12]. This transcriptional activation plays an important role in tumor tissue survival. EPAS-1 can bind to ARNT by its *N*-terminus located in the PAS domain while interacting with other co-factors through its *C*-terminus [12]. A recent study reported that a transcription factor specificity protein-1 (SP-1) binds to EPAS-1 by its zinc-finger domain and potentiates EPAS-1’s transcription factor activity in an HRE-independent manner [13]. This process might be important for venous thromboembolism formation of ovarian clear cell carcinoma patients. SP-1, which specifically recognizes a 5'-GGGCGG-3' motif in promoter, has been proven to be a transcription factor of FBI-1 [14,15,16,17]. Although EPAS-1, as a novel tumorigenesis player, has been investigated for several years regarding its role in hypoxia response of cancer cells, the mechanism it exerts in cancer development has not been demonstrated well. Here we hypothesize that EPAS-1 may participate in tumorigenesis by regulating FBI-1 expression via or with SP-1.

In this study, we found that over-expression of EPAS-1 could up-regulate the expression level of FBI-1, while EPAS-1 knocking-down significantly reduced the level of FBI-1. We found that EPAS-1 could interact with SP-1 and enhanced FBI-1 promoter-luciferase reporter (FBI-1-luc) activity in a SP-1-dependent manner. Further, SP-1 also requires EPAS-1 in inducing FBI-1 expression. By reporter gene assay, we examined 10 SP-1 binding sites and 6 binding sites of other transcription factors in FBI-1 promoter region. We found that EPAS-1 could enhance the SP-1-mediated transcriptional activity specifically. With the obtained information we hypothesized that EPAS-1 participates in FBI-1 related tumorigenesis and cancer development. By CCK-8 assay, cell proliferation assay and clone formation assay we found EPAS-1 could significantly potentiated the proliferation and survival of human lung adenocarcinoma cells. Our work demonstrates a potential mechanism that EPAS-1 regulates FBI-1 expression level via interacting with SP-1, shows the transcription factor-independent role that EPAS-1 may play in tumorigenesis.

## 2. Results and Discussion

### 2.1. Endothelial Pas Domain Protein-1 (EPAS-1) Up-Regulates the Expression Level of Factor Binding IST-1 (FBI-1)

**Figure 1:**
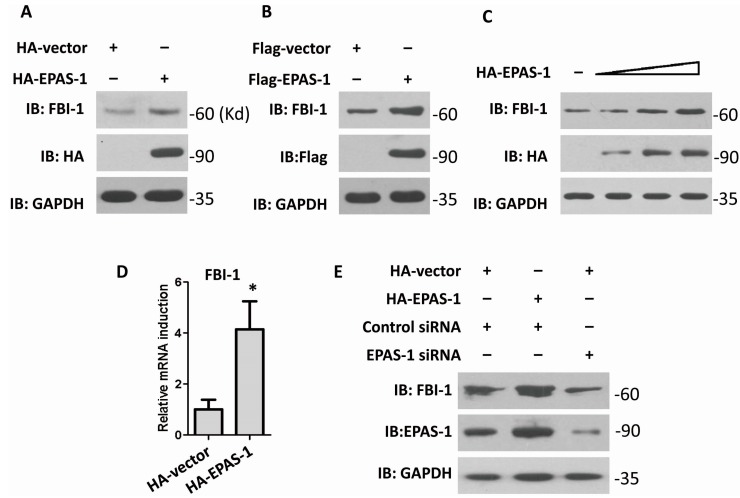
Endothelial pas domain protein-1 (EPAS-1) up-regulates the expression level of Factor binding IST-1 (FBI-1). (**A**,**B**) EPAS-1 over-expression up-regulated FBI-1 protein level. GAPDH were used as loading controls. Molecular sizes were marked in the right of each gel as Kd (same as follow); (**C**) EPAS-1 up-regulated FBI-1 protein level in a dose-dependent manner. GAPDH were used as loading controls; (**D**) EPAS-1 over-expression up-regulated FBI-1 mRNA level. Data points were determined in triplicate and shown with the mean ± SD (* *p* < 0.05, *t*-test); and (**E**) EPAS-1 knockdown reduced FBI-1 protein level. GAPDH were used as loading controls.

To find out whether EPAS-1 participates in regulating FBI-1, HA-EPAS-1 or Flag-EPAS-1 were over-expressed in human HEK293 cells. The result showed that EPAS-1 over-expression elevated FBI-1 protein level (Figure 1A,B). Moreover, the following result showed that FBI-1 could be up-regulated by EPAS-1 in a dose-dependent manner (Figure 1C). To determine whether EPAS-1 acts on the expression level or transcription level of FBI-1, the quantitative real-time PCR was performed. The result showed that over-expression of EPAS-1 could significantly increase the mRNA level of FBI-1 (Figure 1D). Both observations suggested that EPAS-1 specifically up-regulated FBI-1 mRNA level. Moreover, knockdown of EPAS-1 via specific small interfering RNA (siRNA) led to a decrease of FBI-1 in HEK293 cells (Figure 1E). These results indicated that FBI-1 plays a notable role in regulating FBI-1 expression.

### 2.2. Both EPAS-1 and Specificity Protein-1 (SP-1) Control FBI-1 Expression Synergistically

**Figure 2:**
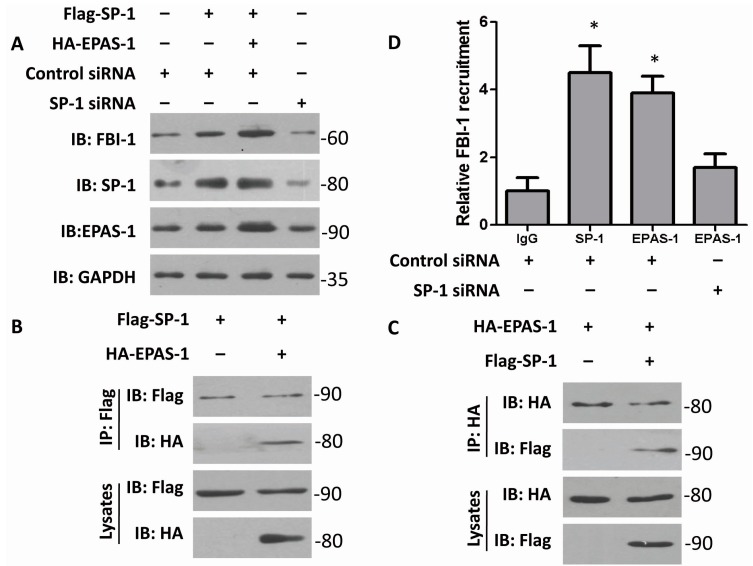
Both EPAS-1 and specificity protein-1 (SP-1) control FBI-1 expression synergistically. (**A**) SP-1 and EPAS-1 double over-expression could further enhance FBI-1 protein level. SP-1 over-expression or EPAS-1 over-expression could not alter each other’s protein level. GAPDH were used as loading controls; (**B**,**C**) EPAS-1 could interact with SP-1; and (**D**) SP-1 mediated the recruitment of EPAS-1 to the FBI-1 promoter. Cells transfected with SP-1 siRNA or control siRNA were prepared and subjected to ChIP by using IgG antibody (negative control) or antibodies for SP-1 and EPAS-1. The immunoprecipitated DNA fragment was quantified by real-time PCR assay. Data points were determined in triplicate and shown with the mean ± SD (* *p* < 0.05, *t*-test).

As previously reported, EPAS-1 can interact with SP-1 to mediate the *FVII*-gene activation in ovarian cancer cells [13]. This funding was different from the canonical understanding that EPAS-1 forms a complex with ARNT and induces hypoxia responsible genes under certain conditions [12]. In order to find out more about EPAS-1’s functional mechanism, the relationship of EPAS-1 and SP-1 in regulating FBI-1 expression was briefly studied in following investigation. Over-expression of SP-1 increased FBI-1 protein level; and both over-expressed SP-1 and EPAS-1 could further increase FBI-1’s expression (Figure 2A). This observation indicated that SP-1 and EPAS-1 at least act synergistically in regulating FBI-1. Moreover, knocking-down SP-1 by specific siRNA reduced the level of FBI-1 (Figure 2A). Together with the previous funding (described in Figure 1), it is possible to conclude that EPAS-1 induces FBI-1’s expression in a SP-1-dependent manner. Then, the interaction of EPAS-1 and SP-1 was examined by co-immunoprecipitation (Figure 2B,C), which suggested that the assembling of EPAS-1/SP-1 complex may be important for FBI-1 regulation. Notably, the CHIP result showed that both SP-1 and EPAS-1 could directly interact with FBI-1 promoter; and when SP-1 was in knockdown, the ability of EPAS-1 interacting with the *FBI-1* gene is weakened (Figure 2D).

### 2.3. EPAS-1 Increases FBI-1-Luc Activity in a SP-1-Dependent Manner

**Figure 3:**
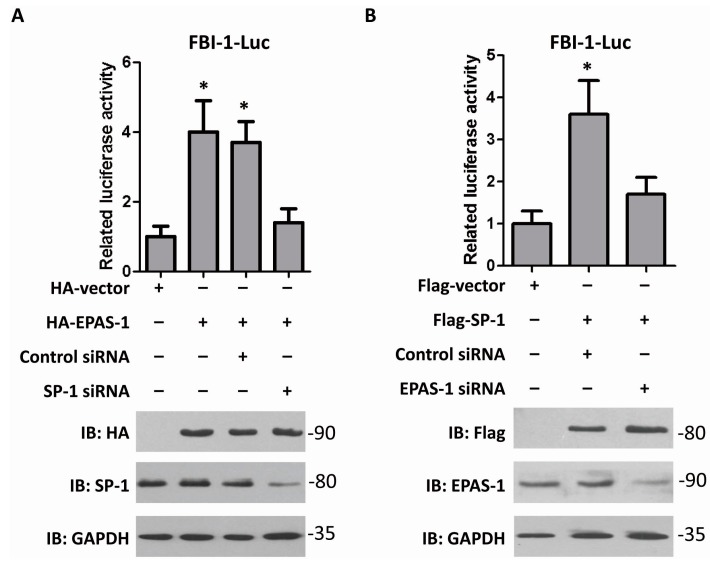
EPAS-1 increases FBI-1-luc activity in a SP-1-dependent manner. (**A**) EPAS-1 over-expression up-regulated FBI-1-luc activity, but SP-1 knockdown restricted this effect. Data points were determined in triplicate and shown with the mean ± SD (* *p* < 0.05, *t*-test); (**B**) SP-1 over-expression up-regulated FBI-1-luc activity, but EPAS-1 knockdown restricted this effect. Data points were determined in triplicate and shown with the mean ± SD (* *p* < 0.05, *t*-test).

To confirm that EPAS-1 up-regulates FBI-1 expression level by potentiating its gene transcription, the reporter gene vector contains FBI-1 promoter region (−2200~+30 bp) was cloned and the dual-luciferase reporter gene assay was performed. The results showed over-expression of EPAS-1 significantly increased FBI-1-luc activity, while over-expression of SP-1 achieved the same conclusion (Figure 3). Moreover, the result showed SP-1 knockdown resisted the raise of FBI-1-luc activity under EPAS-1 over-expression (Figure 3A), which indicated that SP-1 is indispensible for EPAS-1 in regulating FBI-1-luc activity. When knocking-down EPAS-1, the change of FBI-1-luc activity was not significant even when SP-1 was over-expressed (Figure 3B). Together, those results might suggest that EPAS-1 and SP-1 work impartibly in controlling FBI-1 expression.

### 2.4. EPAS-1 Specifically Regulates SP-1-Mediated FBI-1 Expression

**Figure 4:**
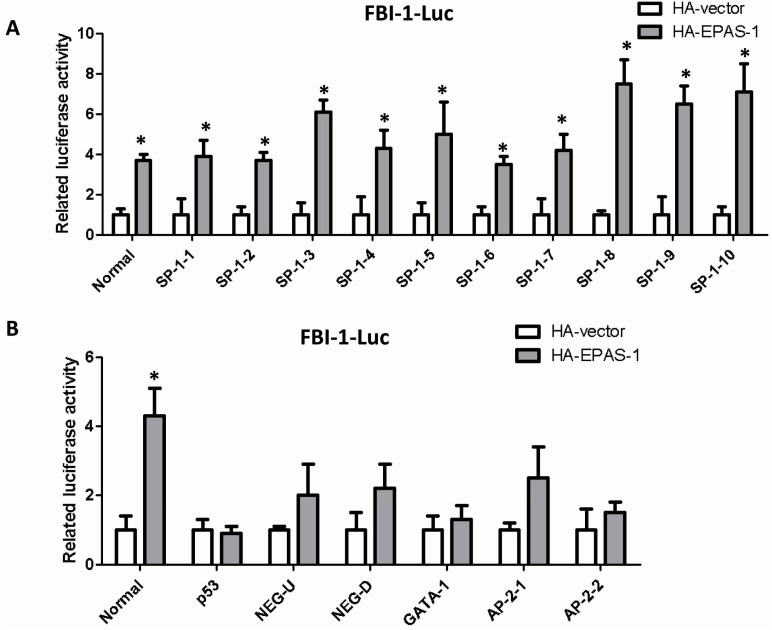
EPAS-1 specifically regulates SP-1-mediated FBI-1 expression. (**A**) EPAS-1 significantly induced the luciferase activities of all the SP-1 binding sites on FBI-1 promoter. Data points were determined in triplicate and shown with the mean ± SD (* *p* < 0.05, *t*-test). The luciferase reporter gene vectors were described in reference [18]; (**B**) EPAS-1 could not significantly induce the luciferase activities of the non-SP-1 binding sites on FBI-1 promoter. Data points were determined in triplicate and showed with the mean ± SD (* *p* < 0.05, *t*-test). The luciferase reporter gene vectors were described in reference [18].

There are several responsible elements for different transcription factors in FBI-1 promoter region [18]. We analyzed the previous report about the promoter region of *FBI-1* gene, cloned 10 SP-1 binding sites and other sites which contain the regulatory elements such as NEG-U and NEG-D or be responsible to other transcription factors such as p53, GATA-1 and AP-2. The gene reporter assay showed that EPAS-1 specifically increased the luciferase activities of the full-length promoter (Normal) and all the SP-1 binding sites vectors (Figure 4A). On the other hand, EPAS-1 could not significantly alter the activities of other reporter gene vectors containing the non-SP-1-targeting sites described above (Figure 4B). Both results indicated that EPAS-1 specifically participates in SP-1-mediated FBI-1 expression.

### 2.5. EPAS-1 Potentiates Human Lung Adenocarcinoma Cell Survival and Proliferation

**Figure 5:**
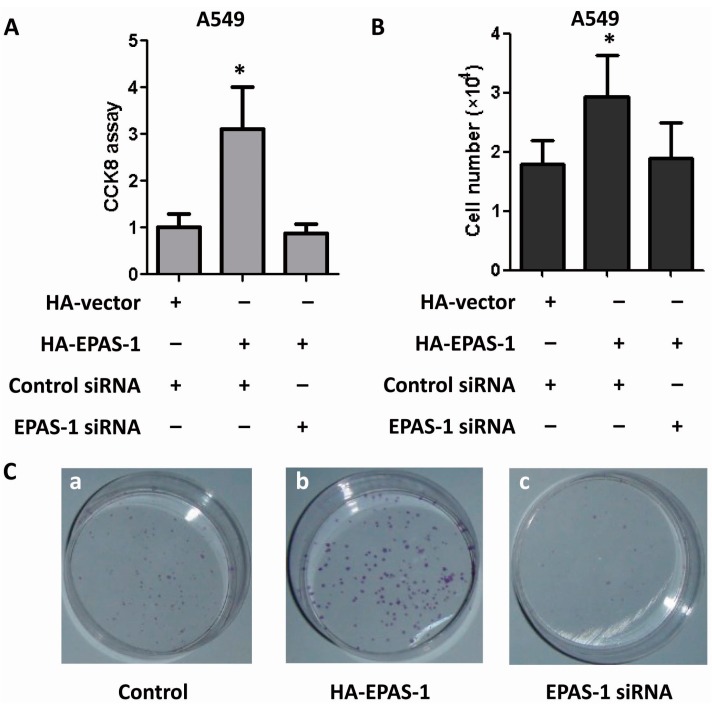
EPAS-1 potentiates human lung adenocarcinoma cell survival and proliferation. (**A**) EPAS-1 over-expression increased A549 cell viability while its knockdown restricted this effect. Data points were determined in triplicate and shown with the mean ± SD (* *p* < 0.05, *t*-test); (**B**) EPAS-1 over-expression increased A549 cell proliferation while its knockdown restricted this effect. Data points were determined in triplicate and shown with the mean ± SD (* *p* < 0.05, *t*-test); (**C**) EPAS-1 over-expression increased A549 cell clone formation ability while its knockdown restricted this effect. (**a**) A549 cells were transfected with control HA-vector and control siRNA; (**b**) A549 cells were transfected with HA-EPAS-1 vector and control siRNA; (**c**) A549 cells were transfected with control HA-vector and EPAS-1 siRNA.

Next we examined the role EPAS-1 plays in human lung adenocarcinoma cell survival and proliferation. Lung adenocarcinoma A549 cells were employed and all investigations suggested that EPAS-1 potentiates their growth, survival and clone formation ability. CCK-8 assay, a method similar to MTT to test cell viability, was performed and the result showed that EPAS-1 over-expression significantly increased A549 cell viability while EPAS-1 knockdown reversed this effect (Figure 5A). Cell number was counted during the studies. EPAS-1 over-expression significantly increased A549 cell number while EPAS-1 knockdown showed no effect on it (Figure 5B). In clone formation assay, EPAS-1 over-expression significantly increased the colony number of A549 cells, but EPAS-1 knockdown reduced the colony number (Figure 5C). Those data indicated that EPAS-1 plays an important role in human lung adenocarcinoma cell survival and proliferation.

## 3. Experimental Section

### 3.1. Plasmids and Antibodies

The HA-EPAS-1, Flag-EPAS-1 and Flag-SP-1 were constructed by PCR, followed by subcloning into various vectors. All the FBS-1 related luciferase vectors were gifts from Yutao Yang. The anti-FBI-1 antibody, anti-Flag antibody and anti-HA antibody were bought from Sigma (St. Louis, MO, USA), the anti-SP-1 antibody, anti-EPAS-1 antibody and anti-GAPDH antibody were purchased from Santa Cruz (Santa Cruz, CA, USA).

### 3.2. Cell Culture and Transfection

HEK293T cells and A549 cells were cultured in DMEM (Corning, Lowell, MA, USA) supplemented with 10% fetal bovine serum (FBS, Corning). Cells were transfected with Lipofectamine 2000 following the manufacturer’s protocol (Invitrogen, Carlsbad, CA, USA). After 48 h of transfection, cells were harvested and lysed in 200 μL of reporter lysis buffer (Promega, Madison, WI, USA). A luciferase assay was carried out using a dual luciferase assay kit (Promega), and the enzymatic activity of luciferase was measured using a luminometer (Promega).

### 3.3. Reporter Gene Assay

To analyze the promoter activities, an empty pGL3-basic vector (Promega) was used as a negative control, and the pRL-TK vector (Promega) was cotransfected as an internal control.

### 3.4. RNA Interference

The RNAs were synthesized by Shanghai GenePharm. All siRNAs were transfected into the cells according to the manufacturer’s protocol.

### 3.5. Co-Immunoprecipitation and Western Blot

For general cell lysis, transfected cells were harvested and lysed in HEPES lysis buffer (20 mM HEPES pH 7.2, 50 mM NaCl, 0.5% Triton, X-100, 1 mM NaF, 1 mM dithiothreitol) and boiledwith 2× SDS/PAGE loading buffer. For immunoprecipitation, cell lyates were prepared in 500 mL HEPES buffer supplemented with protease inhibitor cocktail (Roche, Indianapolis, IN, USA). Immunoprecipitation was performed using mouse anti-Flag (2.5 mg) for 4 h at 4 °C followed by incubation with protein A/G-agarose beads (Santa Cruz) overnight at 4 °C. Beads were then washed three times in HEPES lysis buffer and examined by immunoblotting with the indicated primary antibodies and appropriate secondary antibody, followed by detection with Super Signal chemiluminescence kit (Pierce, Rockford, IL, USA).

### 3.6. CCK-8 Assay and Cell Counting

Cells were seeded on 96-well plates. Then transfected with plasmids or siRNAs for 48 h, culture medium was replaced with fresh medium containing 10 mL CCK-8 solution, and the plate was incubated for 30 min. Cell viability was detected by scanning with a microplate reader at 450 nm.

### 3.7. Clone Formation Assay

Cells were transfected with transfected with plasmids or siRNAs for for 2 weeks, and colonies resistant to G418 (800 mg/mL) selection were stained with crystal violet (0.5% in 20% ethanol) [19].

### 3.8. RNA Extraction and Quantitative Real-Time PCR

Total RNA was isolated from 293T cells using TRIzol reagent according to the manufacturer’s instructions (Invitrogen). Total RNA from each sample was reverse transcribed with random primers using a reverse transcriptase kit (Takara, Dalian, China) followed by quantitative real-time PCR. Primers sequences for FBI-1 are: forward primer, 5'-GGGGACAGCGACGAGGAG-3'; reverse primer, 5'-CGTAGTTGTGGGCAAAGG-3'. The following primers for β-actin were also used: forward primer, 5'-CTCCATCCTGGCCTCGCTGT-3'; reverse primer, 5'-GCTGTCACCTTCACCGTTCC-3'.

### 3.9. Chromatin Immunoprecipitation

The Chromatin immunoprecipitation (ChIP) assay was performed following a protocol provided by the ChIP kit (Upstate, Lake Placid, NY, USA). Cells were fixed by adding formaldehyde to the medium to a final concentration of 1%. After cross-linking, glycine was added to a final concentration of 125 mM, and the cells were then harvested with lysis buffer. The nuclei of the cells were pelleted by centrifugation and re-suspended in nuclear lysis buffer. The nuclear lysates were sonicated to generate to the DNA fragments size of 0.5–1 kb, and then the immunoprecipitation assay was performed with anti-SP-1 or anti-EPAS-1 antibodies, respectively. Real-time PCR amplification was performed with DNA extracted from the immunoprecipitates and primers flanking the SP-1 response elements in the FBI-1 promoter. The primers are: Sense primer, 5'-ACCATTCTCATGCACAGCT-3', Antisense primer, 5'-AGCCTGGGCAACAGAGCAAG-3'.

### 3.10. Statistical Analysis

SPSS software (SPSS Statistics 17.0, IBM, Armonk, NY, USA) was used for the statistical analysis. Student’s *t*-test was performed to evaluate the significance of the differences between test groups and control group.

## 4. Conclusions

In this study, we demonstrate the relationship between EPAS-1 and SP-1 in regulating the expression of proto-oncogene FBI-1. As EPAS-1 plays an important role in tumor cell survival and proliferation via participating indispensably in SP-1 transcription factor-mediated FBI-1 induction, it could be a novel therapeutical target for cancer intervention.

## References

[1] Pessler F., Pendergrast P.S., Hernandez N. (1997). Purification and characterization of FBI-1, a cellular factor that binds to the human immunodeficiency virus type 1 inducer of short transcripts. Mol. Cell. Biol..

[2] Laudes M., Christodoulides C., Sewter C., Rochford J.J., Considine R.V., Sethi J.K., Vidal-Puig A., O’Rahilly S. (2004). Role of the POZ zinc finger transcription factor FBI-1 in human and murine adipogenesis. J. Biol. Chem..

[3] Liu C.J., Prazak L., Fajardo M., Yu S., Tyagi N., D. I., Cesare P.E. (2004). Leukemia/lymphoma-related factor, a POZ domain-containing transcriptional repressor, interacts with histone deacetylase-1 and inhibits cartilage oligomeric matrix protein gene expression and chondrogenesis. J. Biol. Chem..

[4] Maeda T., Hobbs R.M., Pandolfi P.P. (2005). The transcription factor *Pokemon*: A new key player in cancer pathogenesis. Cancer Res..

[5] Widom R.L., Lee J.Y., Joseph C., Gordon-Froome I., Korn J.H. (2001). The hcKrox gene family regulates multiple extracellular matrix genes. Matrix Biol..

[6] Pessler F., Hernandez N. (2003). Flexible DNA binding of the BTB/POZ-domain protein FBI-1. J. Biol. Chem..

[7] Maeda T., Hobbs1 R.M., Merghoub1 T., Guernah1 I., Zelent A., Cordon-Cardo C., Teruya-Feldstein J., Pandolfi1 P.P. (2005). Role of the proto-oncogene Pokemon in cellular transformation and *ARF* repression. Nature.

[8] Lin C.C., Zhou J.P., Liu Y.P., Liu J.J., Yan X.N., Amarsanaa J., Zhang Z.P., Guleng B., Ren J.L. (2012). The silencing of Pokemon attenuates the proliferation of hepatocellular carcinoma cells *in vitro* and *in vivo* by inhibiting the PI3K/Akt pathway. PLoS One.

[9] Cui J., Yang Y., Zhang C., Hu P., Kan W., Bai X., Liu X., Song H. (2011). FBI-1 functions as a novel AR co-repressor in prostate cancer cells. Cell. Mol. Life Sci..

[10] Jeon B.N., Yoo J.Y., Choi W.I., Lee C.E., Yoon H.G., Hur M.W. (2008). Proto-oncogene FBI-1 (Pokemon/ZBTB7A) represses transcription of the tumor suppressor *Rb* gene via binding competition with Sp1 and recruitment of co-repressors. J. Biol. Chem..

[11] Keith B., Simon M.C. (2007). Hypoxia-inducible factors, stem cells, and cancer. Cell.

[12] Majmundar A.J., Wong W.J., Simon M.C. (2010). Hypoxia-inducible factors and the response to hypoxic stress. Mol. Cell.

[13] Koizume S., Ito S., Miyagi E., Hirahara F., Nakamura Y., Sakuma Y., Osaka H., Takano Y., Ruf W., Miyagi Y. (2012). HIF2α-Sp1 interaction mediates a deacetylation-dependent *FVII*-gene activation under hypoxic conditions in ovarian cancer cells. Nucleic Acids Res..

[14] Gidoni D., Dynan W.S., Tjian R. (1984). Multiple specific contacts between a mammalian transcription factor and its cognate promoters. Nature.

[15] Choi W.I., Jeon B.N., Yun C.O., Kim P.H., Kim S.E., Choi K.Y., Kim S.H., Hur M.W. (2009). Proto-oncogene FBI-1 represses transcription of *p21CIP1* by inhibition of transcription activation by p53 and Sp1. J. Biol. Chem..

[16] Suske G. (1999). The Sp-family of transcription factors. Gene.

[17] Cui J., Meng X., Gao X., Tan G. (2010). Curcumin decreases the expression of Pokemon by suppressing the binding activity of the Sp1 protein in human lung cancer cells. Mol. Biol. Rep..

[18] Yang Y., Zhou X., Zhu X., Zhang C., Yang Z., Xu L., Huang P. (2008). Cloning and functional analysis of 5'-upstream region of the *Pokemon* gene. FEBS J..

[19] Zhang P., Ma X., Song E., Chen W.H., Pang H.G., Ni D., Gao Y., Fan Y., Ding Q., Zhang Y., Zhang X. (2013). Tubulin cofactor A functions as a novel positive regulator of ccRCC progression, invasion and metastasis. Int. J. Cancer.

